# Dynamic Imaging of Experimental *Leishmania donovani-*Induced Hepatic Granulomas Detects Kupffer Cell-Restricted Antigen Presentation to Antigen-Specific CD8^+^ T Cells

**DOI:** 10.1371/journal.ppat.1000805

**Published:** 2010-03-12

**Authors:** Lynette Beattie, Adam Peltan, Asher Maroof, Alun Kirby, Najmeeyah Brown, Mark Coles, Deborah F. Smith, Paul M. Kaye

**Affiliations:** Centre for Immunology and Infection, Hull York Medical School and Department of Biology, University of York, York, United Kingdom; University of Wisconsin-Madison, United States of America

## Abstract

Kupffer cells (KCs) represent the major phagocytic population within the liver and provide an intracellular niche for the survival of a number of important human pathogens. Although KCs have been extensively studied in vitro, little is known of their in vivo response to infection and their capacity to directly interact with antigen-specific CD8^+^ T cells. Here, using a combination of approaches including whole mount and thin section confocal microscopy, adoptive cell transfer and intra-vital 2-photon microscopy, we demonstrate that KCs represent the only detectable population of mononuclear phagocytes within granulomas induced by *Leishmania donovani* infection that are capable of presenting parasite-derived peptide to effector CD8^+^ T cells. This restriction of antigen presentation to KCs within the *Leishmania* granuloma has important implications for the identification of new candidate vaccine antigens and for the design of novel immuno-therapeutic interventions.

## Author Summary

*Leishmania donovani* is a protozoan parasite that causes severe disease in humans with associated pathology in the spleen and liver. In experimental models of *L. donovani* infection, the hepatic response to infection is characterised by the presence of a focal mononuclear cell-rich inflammatory response (a granuloma) surrounding cells infected with intracellular amastigotes. Granulomas provide focus to the ensuing immune response, helping to contain parasite dissemination and providing the major effector site responsible for parasites elimination from the liver. Although granulomas are believed to form around infected resident liver macrophages (Kupffer cells), the role of these cells in intra-granuloma antigen presentation is currently unknown. As CD8^+^ T cells have been shown to play an important role in hepatic resistance to *L. donovani* following natural infection, vaccination and during immunotherapy, we asked which cells within the granuloma microenvironment serve as targets for antigen recognition by effector CD8^+^ T cells. Here we provide evidence that the heavily infected mononuclear cell core of the granuloma is composed almost entirely of Kupffer cells, many having migrated from the surrounding sinusoids. Furthermore, by intra-vital 2-photon microscopy, we show that only Kupffer cells laden with intracellular amastigotes are able to form long-lasting antigen-specific interactions with CD8^+^ T cells within the granuloma microenvironment. These data have important implications for the understanding of how granulomas function to limit infection and may have important implications for the development of vaccines to *Leishmania* that are designed to induce CD8^+^ T cell responses.

## Introduction

Kupffer cells (KCs), first identified in 1876, are now recognised as the major population of mononuclear phagocytes to inhabit the resting liver. Lining the sinusoids, KCs express a wide range of phagocytic and innate recognition receptors, including CD32 [1], lectin receptors [2] and TLRs (notably TLR2, 3, 4 and 9) [3], and their avid phagocytic activity has been associated with the clearance of blood borne pathogens and the maintenance of immune homeostasis [4]. Although for many years regarded as a homogenous population, recent data suggest that KCs may be divided into two sub-populations, one sessile and radiation resistant, the other motile and bone marrow derived and expressing higher levels of the costimulatory molecule CD80 [5], reminiscent of the CX3CR1^+^ subset of monocytes that were recently shown to patrol healthy tissues including blood vessels and the skin [6]. In spite of the importance for KCs in the uptake of pathogens, data on their role in the presentation of pathogen-derived antigens is scarce, with most studies focusing on the role of sinusoidal endothelial cells [7] and hepatocytes [8] in the induction of CD8^+^ T cell tolerance, or the ability of hepatic stellate cells and dendritic cells (DCs) to prime CD4^+^, CD8^+^ and NKT cells [9],[10].

In addition to providing a first line of defense against pathogens, KCs are also believed to be involved in downstream events associated with chronic disease, notably in granulomatous inflammation. Granulomas are well-defined mononuclear cell-rich aggregates that ideally serve to ‘contain and control’ pathogen spread [11],[12], but when unregulated may also contribute to disease pathology [13]. Experimental infection with visceralising species of *Leishmania* provides, along with experimental mycobacterial infection, some of the best characterised models for evaluating granuloma form and function [14],[15], particularly within the hepatic microenvironment. In experimental visceral leishmaniasis (VL), current models of hepatic granuloma formation, based largely upon data obtained using static imaging approaches, suggest that infected KCs create the central nidus of the granuloma, fusing with other mononuclear phagocytes of less well-defined origin, and ultimately attracting lymphocytes and monocytes [16] through chemokine secretion [17],[18]. More recent studies using BCG infection have provided some additional information on macrophage dynamics and T cell motility within hepatic granulomas during this infection [19] but fail to directly address KC function. In spite of the fact that granuloma macrophages harbour much of the hepatic pathogen load during experimental VL, and there have been numerous reports of intracellular infection with *Leishmania* parasites affecting macrophage APC function [20],[21],[22] the role of KCs as antigen presenting cells in these models has yet to be directly addressed.

In experimental VL, CD8^+^ T cell responses are required for the effective clearance of parasites [23], provide one of the best correlates of protection following vaccination [24] and can be used effectively in adoptive immunotherapy [25]. These and other data [26],[27],[28] have fuelled interest in the potential for immuno-prophylactic or immuno-therapeutic expansion of CD8^+^ T cells as a means of disease control. In the present study, therefore, we have directly addressed the question of whether KCs laden with intracellular *Leishmania* can be directly recognized by antigen-specific effector CD8^+^ T cells. Our data demonstrate that the majority of amastigote-infected cells within the core of a granuloma represent KCs that have migrated from neighbouring sinusoids, and by flow cytometry, only this population of KCs expresses detectable K^b^-SIINFEKL complexes after infection of mice with OVA-transgenic *L. donovani.* To determine whether KCs engage in cognate interactions with CD8^+^ T cell *in situ*, we used intra-vital 2-photon microscopy to quantify T cell recruitment into and behaviour within individual granulomas. These studies show that effector CD8^+^ T cells accumulate in granulomas in an antigen-specific manner, as a result of having prolonged interactions with amastigote-laden KCs. Thus, we provide the first evidence that KCs undergo cognate interactions with CD8^+^ T cells in the context of *Leishmania* infection, a result which has important implications for the development of immunotherapy against this intracellular pathogen.

## Results

### Distribution of intracellular amastigotes in the *L. donovani*-infected liver

**Figure 1:**
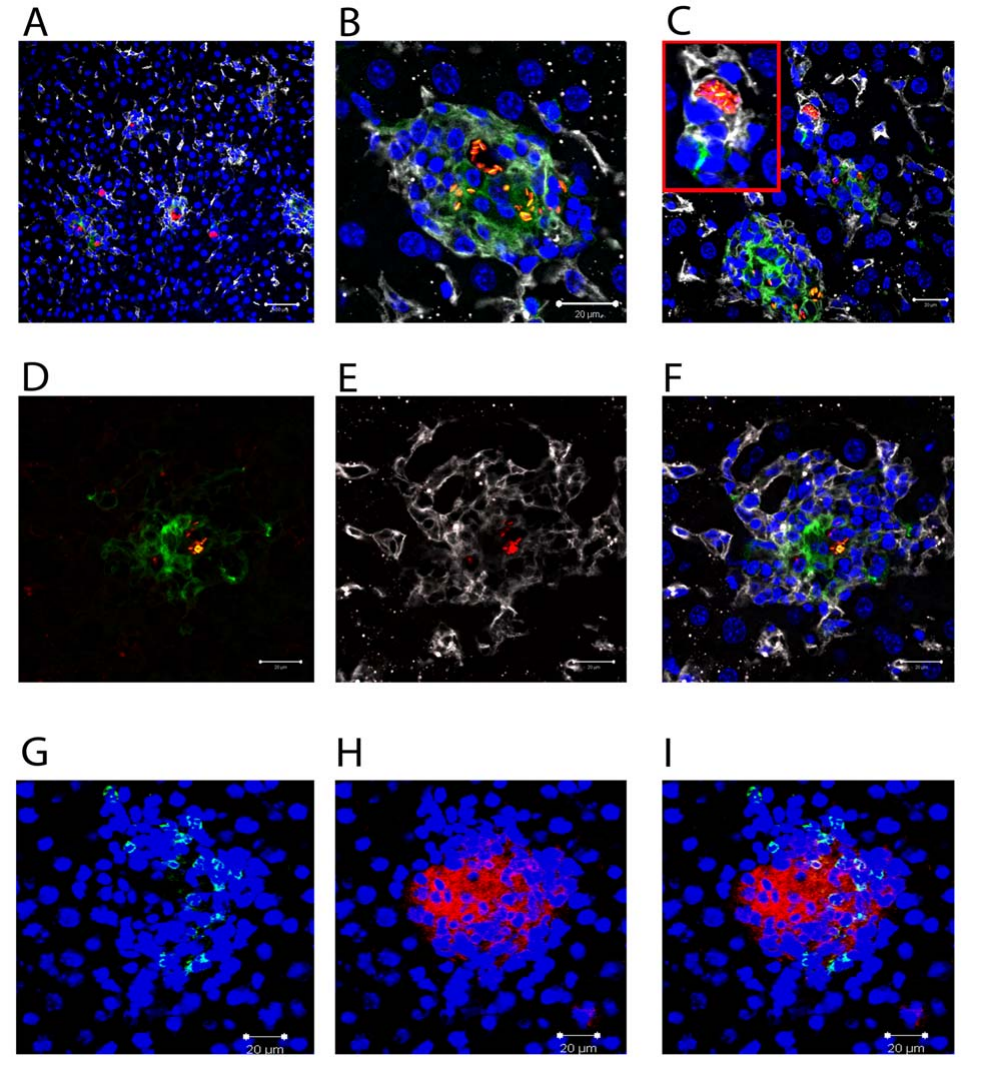
Distribution of *Leishmania* amastigotes in the liver. C57BL/6 mice were infected with tdTom-*L. donovani* (A–F) or WT-*L. donovani* amastigotes (G–I) and 14 days later liver tissue was processed for confocal microscopy. **A**)**–F**) Intracellular amastigotes (red) are shown in combination with staining for F4/80 (white) and CD11c (green). **A**) Low magnification view to show diversity of the granulomatous response. **B**) High magnification image of single granuloma with macrophages containing numerous amastigotes. **C**) Infected F4/80^+^ cells with limited inflammatory cell recruitment. **D**) CD11c (green) and **E**) F4/80 (white) expression on amastigote (red) infected cells at the granuloma core. **F**) overlay of D) and E). **G**) CD11b^+^ cells (green) and **H**) F4/80^+^ cells (red) were predominantly localised to distinct sites within the granuloma and F4/80^+^ cells at the core did not express CD11b (**I**, overlay). DAPI was used as a nuclear counterstain (blue). Scale bars 50 µm for A) and 20 µm for B–F).

*L. donovani* amastigotes are usually identified in tissue based on their characteristic staining pattern after H&E staining of thin sections [14], with the sensitivity of detection, particularly for individual parasites being improved by immuno-histology using polyclonal or monoclonal antibodies [29]. To more readily observe parasites by fluorescent microscopy, we generated stable infective clones of *L. donovani* expressing tdTomato (tdTom; [30]), a fluorochrome amenable to both confocal and multi-photon imaging. We first infected mice with tdTom-*L. donovani* and examined their distribution in the liver at day 14 p.i. (**Figure 1**) in conjunction with staining for F4/80, a marker of mature KCs [31] and CD11c, a marker characteristically associated with DCs [32]. *L. donovani* amastigotes were readily apparent both at low magnification, where individual amastigotes within heavily-infected cells could not be resolved (**Figure 1A**), and at higher magnification, where individual parasites were easily distinguished (**Figure 1B**). Parasites were observed in two main anatomical locations: within granulomas, where they were predominantly associated with the core, and within the parenchyma, where by DAPI staining they appeared to be within isolated cells in areas largely devoid of local inflammatory reactions (**Figure 1C**). Almost invariably, amastigotes in either location were found within F4/80^+^ cells (**Figure 1A–C**). The close apposition and membrane interdigitation of F4/80^+^ cells made it difficult to score individual cells, so we did not attempt to calculate the percentage of F4/80^+^ cells that were infected within the core of the granuloma. Reminiscent of the pattern of staining with NLDC-145, a DEC 205-specific antibody [33], a diffuse but detectable level of CD11c expression was also observed on cells at the core of many, but not all, granulomas. These CD11c^+^ cells also expressed somewhat lower levels of F4/80, compared to the F4/80^+^ CD11c^−^ cells that occupied the granuloma mantle (**Figure 1D–F**). Heterogeneity of expression of CD11c within granulomas did not correlate with the presence or absence of amastigotes. In contrast, CD11b^+^ cells were usually found in the granuloma mantle, with some clearly identifiable as neutrophils based on nuclear morphology. Importantly, the large amastigote-laden cells at the granuloma core that co-stained for F4/80 and CD11c were almost uniformly CD11b^−^ (**Figure 1G–I**). These data, together with previously published studies [33] suggest that the majority of intra-granuloma amastigotes are found within F4/80^+^ cells, and some of these cells acquire markers in this local micro-environment that are often associated with DC.

Although flow cytometry might be expected to provide a means for further phenotypic analysis of tdTom-*L. donovani* infected macrophages, separation of tdTom-*L donovani* positive cells by cell sorting (**Figure S1A and B**), followed by cytospin and Giemsa staining (**Figure S1C**) indicated that parasites became associated with a range of different cell types, including macrophages, monocytes, lymphocytes and polymorphonuclear cells. In many cases, parasites were bound rather than internalised by these cells. Similarly, co-preparation of cells after mixing of liver tissue from C57BL/6 (CD45.2) mice that were infected with WT-*L. donovani* and from B6.CD45.1 mice that were infected with tdTom-*L. donovani* clearly demonstrated transfer of tdTom-*L.donovani* from CD45.1 to CD45.2 cells. Hence, flow cytometry does not provide a reliable means to further characterise the phenotype of cells infected in situ.

### The macrophage rich core of the *L. donovani* granuloma is the result of recruitment of resident Kupffer cells

**Figure 2:**
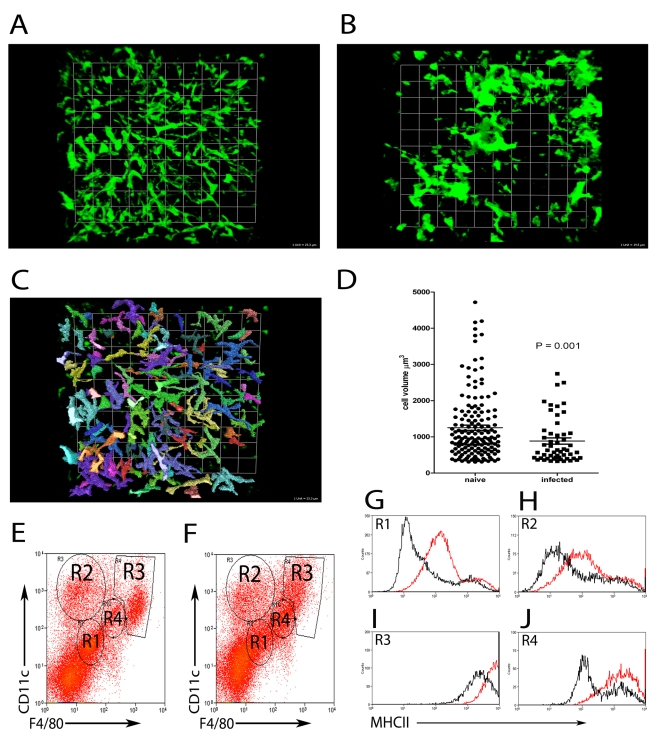
Kupffer Cell redistribution as a result of *L. donovani* infection. **A**) Whole mount immuno-histochemistry showing the distribution of F4/80^+^ (green) KCs in naïve (1 unit  = 23.3 µm) and **B**) *L. donovani* infected liver (1 unit  = 19.5 µm). **C**) Image from A) demonstrating the method used to determine the volume of F4/80 positive cells with Volocity software. **D**) Comparison of cell volumes of single F4/80^+^ cells in naïve and non-granuloma associated KC in infected livers (mean ± SEM). **E**) Hepatic mononuclear cell preparations showing 4 populations of cells based on expression of F4/80 and CD11c from naïve and **F**) day 14 infected mice. **G–J**) expression of MHCII on the surface of R1 (**G**), R2 (**H**), R3 (**I**) and R4 (**J**) populations from naïve (black lines) and infected (red lines) mice. Data is representative of 21 replicates from 5 individual mice.

Although macrophages are acknowledged to be a central feature of granulomatous inflammation, the precise origin of these cells has not been directly determined. To address this issue, we first studied the distribution of liver resident and inflammatory phagocytes in naïve and *L. donovani*-infected mice. KCs in the liver of uninfected mice show a characteristically uniform distribution, lining the sinusoids and forming a reticular surveillance network [34]. To more fully determine the spatial context in which KCs line the sinusoids, we performed whole mount immuno-histochemistry, using F4/80 as a marker of mature KCs (**Figure 2**). In naïve mice, large KCs with extensive projections were readily apparent within sinusoidal spaces (**Figure 2A** **, and Video S1**) forming a regular uniformly distributed phagocytic network. In contrast, in mice infected for 14 days with *L. donovani*, many KCs were aggregated within granulomas, leaving large areas of the sinusoidal network devoid of detectable KCs (**Figure 2B** **and Video S1**). Strikingly, although not participating in the granulomatous inflammatory response, KCs that remained isolated within the sinusoidal network nevertheless displayed morphological changes, which could be quantified as a reduced total cell volume compared to KCs in uninfected mice (**Figure 2C, D**). Although losing the spatial information provided by whole mount immunohistochemistry, we isolated hepatic mononuclear cells and labeled with F4/80 and CD11c to identify four populations of cells in both naive (**Figure 2E**) and *L. donovani* infected (**Figure 2F**) livers. While all four populations were present in both naïve and infected mice, the proportions changed with infection. CD11c^−^F4/80^−^ cells (**Figure 2E and F, R** **1**) accounted for 51.7+/− 5.13% of F4/80^+^ cells in naïve mice and 38.88 +/− 4.34% in infected mice. CD11c^hi^F4/80^int^ cells (**Figure 2E and F, R** **2**) accounted for 17.11 +/− 3.12% in naïve mice and 19.29 +/− 3.31% in infected mice. CD11c^hi^F4/80^hi^ cells (**Figure 2E and F, R** **3**) accounted for 13.39 +/− 2.51% in naïve mice and 13.5 +/− 2.96% in infected mice. Finally, CD11c^int^F4/80^int^ cells (**Figure 2E and F, R** **4**) accounted for 7.83 +/− 0.87% in naïve mice and 14.67 +/− 4.82% in infected mice. MHCII expression, used as a surrogate marker for macrophage activation, was shown to be upregulated on all four populations upon infection (**Figure 2G–J**). These data suggest that most KCs in the infected liver, even if not recruited into granulomas, had responded to the developing inflammatory environment.

**Figure 3:**
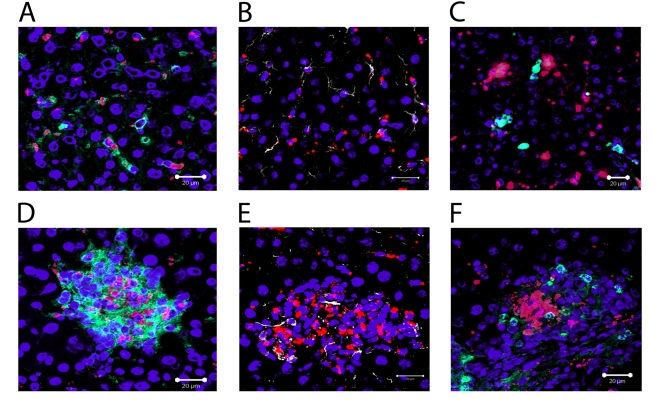
Recruitment of local Kupffer Cells results in a redistribution of nanobeads. Distribution of nanobeads (red) in naïve liver following pre-injection showing **A**) F4/80^+^ Kupffer cells (green), **B**) Desmin^+^ stellate cells (white) and **C**) CD11b^+^ monocytes (green). Distribution of nanocrystals (red), in liver of mice pre-injected with NBs and then infected with *L. donovani*, in **D**) F4/80^+^ Kupffer cells (green), **E**) Desmin^+^ stellate cells (white) and **F**) CD11b^+^ monocytes (green). DAPI was used as a nuclear counterstain (blue). Scale bars for A–F 20 µm.

To determine if the aggregation of KCs in granulomas was due to a re-distribution of liver-resident KCs, or whether this reflected the recruitment/differentiation of blood or BM-derived precursors after infection had been established, we used fluorescent nanobeads (NBs) to label KCs (and other potential liver-resident phagocytic cells) prior to infection. Such cells could then be subsequently discriminated from inflammatory phagocytes recruited after infection (**Figure 3A–F**). We first analysed the distribution of these NBs after intravenous injection into naïve mice. As shown in **Figure 3A**, NBs were readily ingested by liver-resident F4/80^+^ KC in uninfected mice, providing a readily detectable measure of their phagocytic activity. Most KCs were phagocytic (∼74%, n = 42), with a variable phagocytic load of NBs. Within individual KCs, multiple ‘patches’ of NB labeling could often be observed, presumably reflecting uptake of NBs into discrete phagosomes. These patches also varied in size, a result that might reflect either aggregation of NBs during injection and/or coalescence of multiple phagosomes each containing small numbers of NBs. NBs were also phagocytosed by desmin^+^ hepatic stellate cells in naïve mice (∼66% of desmin^+^ cells contained NBs, n = 90), but large aggregates were rarely observed in these cells (**Figure 3B**). CD11b^+^ cells are rare in the resting liver as determined by immuno-histochemistry [35], and when observed, these cells did not contain NBs (**Figure 3C**). We then injected mice with NBs and 4–12 h later, infected them with *L. donovani*. The distribution of NB^+^ cells was then observed at both day 14 p.i. (**Figure 3D–F**) and at d28 p.i. (data not shown), with similar results being obtained at each time point. NBs were readily observed in *L. donovani*- infected mice, confirming their value as a long-term cell tracer. NBs were highly concentrated in granulomas, largely at the core, and almost exclusively within F4/80^+^ KCs (**Figure 3D**). In contrast, although occasionally present within granulomas, hepatic stellate cells were normally excluded from the core of the granuloma and usually did not contain readily distinguishable NBs (**Figure 3E**). Strikingly, NBs were also not observed in CD11b^+^ cells (presumptive monocytes, DC and neutrophils) either at the core of the granuloma or when more peripherally dispersed at the granuloma mantle (**Figure 3F**). To confirm that the distribution of NBs in granulomas was not the result of rapidly recruited inflammatory cells, NBs were injected and the mice infected with *L. donovani* 12 hours later as described above. No significant infiltration of inflammatory cells was observed 6 hours after infection, with the proportions of CD11b^−^, CD11b^int^ and CD11b^hi^ cells being similar between mice that received NBs only or mice that received NBs and *L. donovani*, whether measured in terms of either the frequency or absolute number of cells (**Figure S2**). These data suggest that NB distribution after infection reflects KC redistribution and is not influenced by rapidly recruited inflammatory cells. Collectively, these data therefore strongly support the contention that the core of the granuloma is derived almost exclusively from resident KCs recruited from the sinusoids early during the inflammatory process.

### Macrophages recovered from granuloma-containing liver express MHCI-peptide complexes

**Figure 4:**
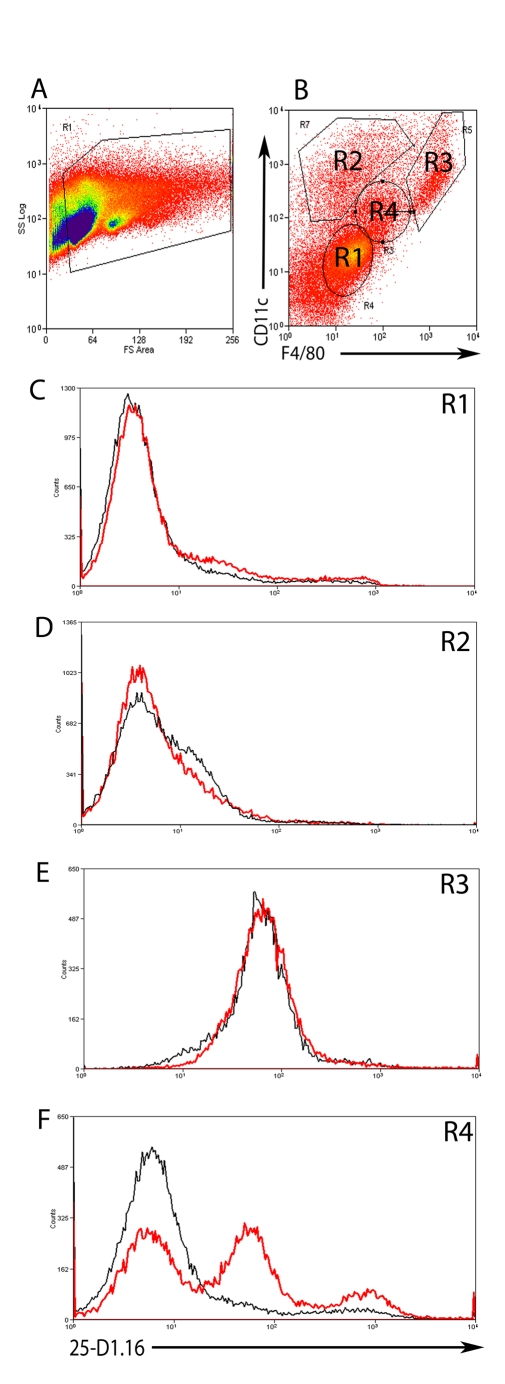
Expression of MHCI-peptide complexes in the liver. **A**) Hepatic mononuclear cell suspensions were gated on FSC and SSC and **B**) CD11c and F4/80 expression to identify 4 different cell populations (R1-4). **C**) Expression of K^b^-SIINFEKL complex as detected by binding of 25-D1.16 on F4/80^−^ CD11c^−^ (R1), **D**) F4/80^int^ CD11c^hi^ (R2) **E**) F4/80^hi^ CD11c^hi^ (R3) and **F**) F4/80^int^ CD11c^int^ (R4) cells from WT *L. donovani* (black line) or PINK (red line) infected mice. Data is representative of two-independent experiments.

As a first step to determining whether cells within hepatic granulomas could present MHC class I-restricted antigens derived from *L. donovani* amastigotes, we infected mice with double transgenic *L. donovani* made by transfecting an OVA-expressing *L. donovani* clone (PINK; [25]) with tdTom. OVA expressed by PINK is localised to the parasite plasma membrane by virtue of the HASPB N-terminal dual acylation sequence [36], and is available for *in vivo* recognition by K^b^- restricted OVA_257–263_ (SIINFEKL) -specific TCR transgenic CD8^+^ T cells [25],[37]. To determine which cells could process and present SIINFEKL derived from these transgenic parasites, we first used 25-D1.16, a mAb specific for this MHC-peptide complex [38]. By immunohistochemistry, however, we were unable to detect expression of this complex in any cells within the infected liver (data not shown), probably reflecting the very low levels of complex expressed in this physiological setting. Although loosing the spatial information provided by immuno-histochemistry, we next used flow cytometery as a more sensitive assay to detect whether this complex was expressed and on which cells (**Figure 4**), comparing the expression of 25-D1.16 on hepatic mononuclear cells isolated from mice infected with either PINK or WT *L. donovani*. Four discrete populations were identified on the basis of CD11c and F4/80 expression (**Figure 4A and B** **, gates 1–4**). In comparison to ‘control’ staining determined from analysis of mice infected with WT *L. donovani*, no expression of 25-D1.16 was observed in CD11c^−^F4/80^−^ cells ((**Figure 4C, R** **1**) nor in CD11c^hi^F4/80^int^ cells (**Figure 4D, R** **2**). These CD11c^hi^F4/80^int^ most likely equate to the small number of intra-granuloma DCs observed by histology (**Figure 1**). We also could not detect specific staining in CD11c^hi^F4/80^hi^ cells (**Figure 4E, R** **3**) though the high autofluorescence of these cells may have precluded detection of low levels of 25-D1.16 expression. In contrast, CD11c^int^F4/80^int^ cells from mice infected with PINK, which represented 11.47±1.4% of total hepatic leucocytes, contained two populations of cells with differing intensity of expression complexes recognised by 25-D1.16 (**Figure 4F, R** **4**). Importantly, CD11c^int^F4/80^int^ cells mice infected with WT *L. donovani* (a genetic control for non-specific mAb binding) were not stained with 25-D1.16. CD31^+^ liver sinusoidal endothelial cells account for approximately 35% of the total hepatic mononuclear cells, but are negative for F4/80 and CD11c. Similarly, hepatic stellate cells, noted for their strong autofluorescence and high side scatter properties [39] make up approximately 3% of the hepatic mononuclear cells in these preparations and are likely located within the R3 population, based on expression of F4/80 and CD11c expression (data not shown). Neither the F4/80^−^ nor the R3 population however expressed MHCI-peptide complexes as determined by 25-D1.16 staining. These data argue, therefore, for expression of the K^b^-SIINFEKL epitope on restricted population(s) of *L. donovani*-infected hepatic cells whose phenotype as determined by flow cytometry closely resembles that of infected F4/80^+^CD11c^lo^ KCs at the core of the granuloma (**Figure 1**).

### Intra-granuloma T cells visualised by 2-photon imaging

Although we detected MHC-peptide complex on presumptive intra-granuloma KCs, the inherent loss of spatial information associated with flow cytometry prompted us to seek alternate approaches to identify antigen recognition by CD8^+^ T cells *in situ*. As real-time imaging of T cell dynamics has been shown to be a valuable tool for analysing T cell-APC [40] and T cell-target [41] interactions, and we had already established an adoptive transfer model that provided indirect evidence for cognate antigen recognition by CD8^+^ T cells, we combined these approaches to study the dynamics of CD8^+^ T cells in the liver of *L. donovani*-infected mice.

**Figure 5:**
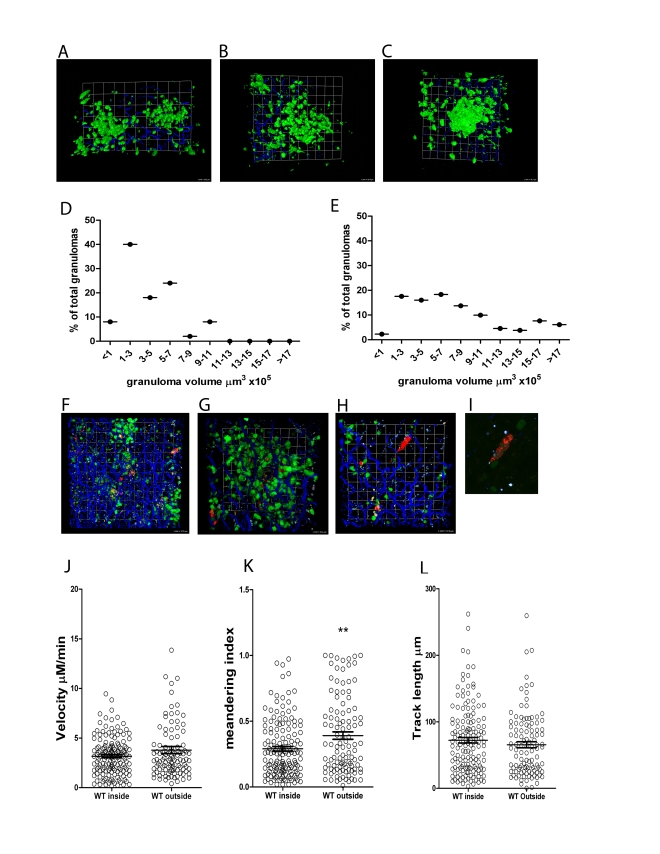
3D imaging of hepatic granulomas. **A–C**) Snapshots of the 3D view of Z-stacks collected from the livers of d14 *L. donovani*-infected hCD2.GFP reporter mice showing the T cell content of individual granulomas. Scale A) 24 µm, B) 20 µm C) 15.7 µm. **D**) Volume distribution for granulomas in the liver of d14 infected (n = 50 granulomas) and **E**) d25 infected hCD2.GFP reporter mice. (n = 131 granulomas) **F–H**) Distribution of tdTom *L. donovani* amastigotes in the liver of d14 infected hCD2.GFP reported mice showing the focal accumulation around some, but not all parasite infected cells. Scale F) 28 µm, G) 16 µm, H) 17.4 µm. **I**) Enlarged view of parasite infected cell in H) to show resolution of single parasites. J–K) OT-I T cell migration within and outside of granulomas was determined in WT *L. donovani* infected livers by calculating cell velocity (J) meandering index (K) and track length (L). ** P<0.01 Bars represent mean +/− SEM.

First, to establish the nature of the T cell environment into which adoptively transferred cells would be imaged, we used hCD2.GFP reporter mice [42] to visualise the entire T cell (and NK cell) content of the *L. donovani* granuloma. In mice infected with either wild type *L. donovani* or tdTom-*L. donovani*, prominent accumulations of T cells were observed from d14 onwards (**Figure 5** and data not shown). These accumulations were heterogeneous in nature with the T cells demarcating a structure that varied from being a large flat accumulation of cells close to the collagenous liver capsule (**Figure 5A, B** **and Video S2**) to more compact, rounded accumulations of cells that protruded further into the parenchyma (**Figure 5C** **and Video S2**). Examination of the total volume of the T cell accumulations at d14 (**Figure 5D**) and d25 (**Figure 5E**) showed that while the response was heterogeneous in nature throughout the time course of infection studied, smaller granulomas were more frequent in early infection, while larger accumulations were seen later in the response. Most T cell accumulations had readily detectable parasites (**Figure 5F and G** **and Video S3**), confirming that these accumulations were indeed granulomas, though as shown earlier using DAPI staining (**Figure 1A**), infected macrophages could also be found in the parenchyma in the absence of local T cell recruitment (**Figure 5H and I** **and Video S3**). Second harmonic imaging of collagen (**Figure 5A–C and F–I**) also confirmed earlier reports indicating that the *L. donovani* granuloma is not highly fibrotic in mice [**43**],[**44**] and in some instances migration of T cells along collagen fibres within individual granulomas was observed (data not shown).

As the tracking of T cells inside BCG-induced granulomas has suggested that the granuloma microenvironment inhibits the motility of T cells in a non-antigen specific manner [19], we compared the dynamics of OT-I T cells found within WT *L. donovani*- induced granulomas with those found in the liver parenchyma of the same infected mice. As shown in Figure 5J-L, we found no significant difference in cell velocity or track length whether cells were moving in the parenchyma or within granulomas. Although the meandering index was higher for cells outside of granulomas, this might reflect the influence of the sinusoidal network on the path of the cell movement. Additional analysis of the instantaneous velocities of cells shown in **Video S5**, also failed to show any obvious difference in the pattern of instantaneous velocity for OT-I cells inside compared to outside of granulomas (**Figure S3**). Hence, unlike the BCG granuloma, the *L. donovani* induced granuloma does not appear to pose a major physical barrier to CD8^+^ T cells motility.

### Antigen-specific CD8^+^ T cells accumulate within *L. donovani*-induced granulomas

**Figure 6:**
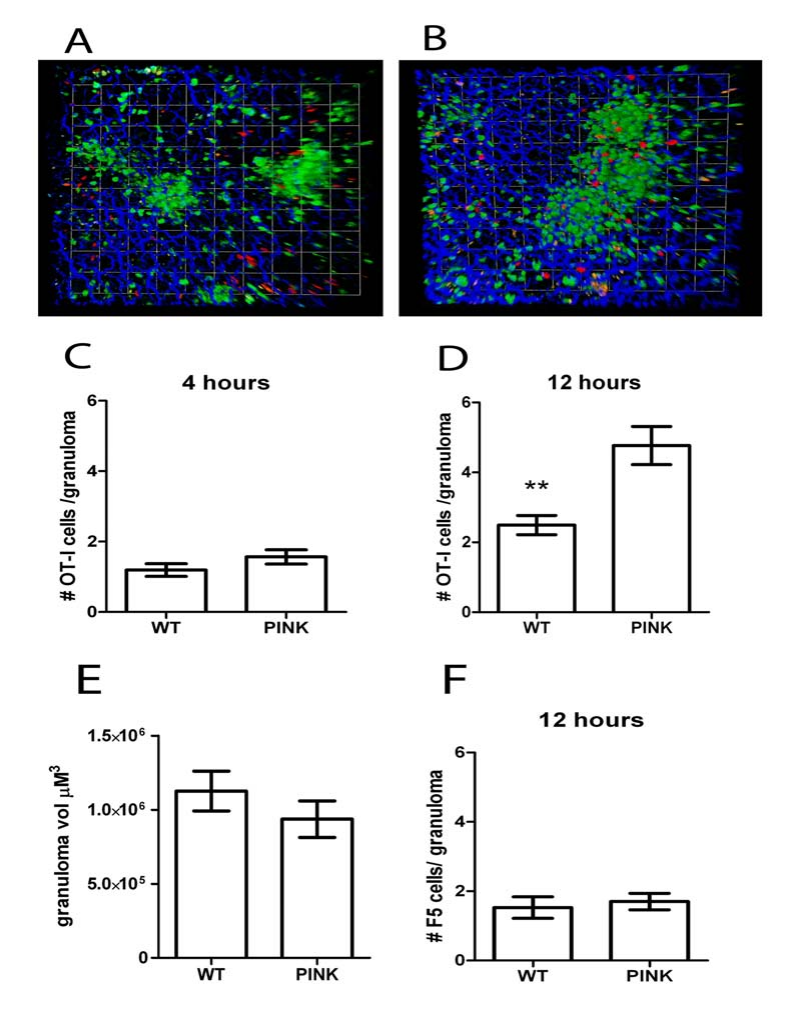
Accumulation of antigen-specific CD8^+^ T cells within hepatic granulomas. **A**) Snapshot of the 3D view of Z-stacks collected from the livers of d21 OVA expressing PINK-infected hCD2.GFP reporter mice that received 10^7^ memory-like CMTMR labelled OT-I T cells (red) 4 h or **B**) 12 h previously. **C**) Quantification of the number of OT-I T cells located within granulomas from d21 *L. donovani* infected mice 4 h after transfer (n = 68 granulomas and 81 cells for WT and 76 granulomas, 19 cells for PINK) or **D**) 12 h after transfer (n = 116 granulomas and 289 cells for WT and 103 granulomas and 491 cells for PINK, ** P<0.001). **E**) 3D volume of granulomas in the livers of d21 hCD2.GFP mice used in (A–D). **F**) Number of F5 T cells within the granulomas of d21-infected WT *L. donovani*- and PINK-infected mice 12 h after transfer (n = 53 granulomas and 81 cells for WT and 57 granulomas and 97 cells for PINK.) Data represents mean ± SEM and is representative of 3 independent experiments.

To study the antigen-specific behaviour of CD8^+^ T cells in granulomas, we labelled effector memory-like CD62L^lo^ OT-I T cells [45] with CMTMR and adoptively transferred these cells into hCD2.GFP mice infected 21d earlier with either WT *L. donovani* or PINK. The fate of these OT-I T cells in the liver was then followed for up to12 h post transfer. Within 4 h of transfer, transferred OT-I T cells were detected in the liver and found to be primarily within sinusoids (**Figure 6A** **and Video S4**) but by 12 h post-transfer, large numbers were fully embedded within granulomas (**Figure 6B** **and Video S4**). From full 3D-reconstructions of granulomas, we scored the number of OT-I T cells embedded within granulomas in mice infected with either WT *L. donovani* or PINK at either 4 h or 12 h post transfer. Although antigen-independent accumulation of OT-I T cells was observed at 4 h (**Figure 6C**), by 12 h, antigen-specific accumulation of OT-I T cells was evident (**Figure 6D**). Importantly, granuloma volume, a surrogate measure of the number of T cells, was not significantly different in mice infected with these two parasite lines, ruling this out as one possible explanation for the effect observed (**Figure 6E**). As an alternate means to confirm the antigen specificity of intra-granuloma CD8^+^ T cell accumulation, we also transferred effector memory-like influenza-specific F5 CD8^+^ T cells into WT *L. donovani* and PINK infected mice. No difference in F5 T cell accumulation was observed in the granulomas in these mice (**Figure 6F**).

### The presence or absence of cognate antigen determines the dynamics of CD8^+^ T cell motility in *L. donovani*-induced granulomas

**Figure 7:**
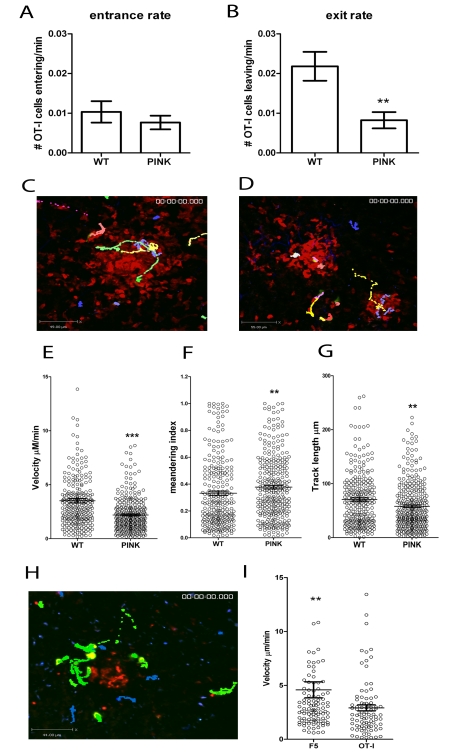
CD8^+^ T cell dynamics in the liver following *L. donovani* infection. **A**) The entrance and **B**) exit rate of CFSE labelled memory-like OT-I T cells 5–14 h post-transfer into d14–21 infected hCD2.GFP mice calculated by dividing the number of OT-I cells entering or leaving each granuloma for each imaging session and dividing by the time of each imaging session to give a rate/min (n = 60 imaging sessions for WT and 71 for PINK infected mice, ** < 0.001). **C**) Snapshot of the extended focus view of a time-lapse imaging sequence showing the cell tracks of CFSE labelled memory-like OT-I T cells transferred into d14-21 WT *L. donovani* or **D**) PINK-infected mice. Comparison of the **E**) cell velocities, **F**) meandering index and **G**) track length of memory-like OT-I T cells transferred into d14-21 WT *L. donovani-* or PINK-infected mice (n = 266 for WT and 311 for PINK, *** P<0.0001, ** P<0.001). **H**) Snapshot of the extended focus view of a time-lapse imaging sequence showing the cell tracks of Hoechst labelled memory-like OT-I T cells (blue tracks) and CFSE labelled memory-like F5 cells (green) transferred into d14-21 PINK infected mice. **I**) Comparison of the cell velocities of memory-like F5 and OT-I T cells transferred into d14-21 PINK-infected mice (n = 105 for F5 and 87 for OT-I T cells). Data represents mean ± SEM, ** P<0.001.

Altered accumulation of CD8^+^ T cells within granulomas could be the result of altered rates of immigration or emigration. To distinguish between these possibilities, we examined the dynamics of OT-I T cell movement within individual granulomas in WT *L. donovani* and PINK-infected mice 5–14 h post-transfer of OT-I T cells. We calculated the rate at which OT-I T cells entered granulomas by dividing the number of cells entering or exiting the granuloma in each imaging period by the length of the imaging period in minutes. No significant differences were seen in the rate at which OT-I T cells entered granulomas in WT *L. donovani*- and PINK-infected mice (**Figure 7A**). In contrast, the rate at which OT-I T cells left granulomas in PINK-infected mice was slower than in WT *L. donovani*-infected mice (**Figure 7B**). The finding that exit rate, but not entrance rate, was influenced by the presence or absence of cognate antigen suggested that OT-I T cells behaved differently if antigen was available. To determine whether this was reflected in altered velocity, we calculated the average velocity of OT-I cells (n = 311 cells from 43 imaging fields) in PINK-infected and OT-I cells (n = 266 cells from 48 imaging fields) in WT *L. donovani-*infected hCD2.VaDs Red mice (here used to identify the border of the granuloma by endogenous labelling of all other T cells). The results of this analysis demonstrated that OT-I T cells moved significantly more slowly in the presence of cognate antigen (**Figure 7C, D, E** **and Video S5**). The meandering index (calculated by diving the displacement of the cell from its original starting point by the total track length of that cell) was significantly higher for OT-I T cells transferred into PINK-infected mice than those transferred into WT *L. donovani-* infected mice (**Figure 7E**). This was reflected by significantly lower track lengths for OT-I cells transferred into PINK infected mice and therefore in the presence of cognate antigen (**Figure 7F**).

As further independent confirmation that the difference in dynamics of OT-I in the presence and absence of antigen was due to antigen recognition and not due to other differences in the granulomas formed following infection with PINK and WT *L. donovani*, we labelled OT-I T cells with hoescsht-33342 and F5 T cells with CFSE and co-transferred equal numbers into PINK-infected mice. In these experiments, granulomas were visualised by pre-injection of fluorescent NBs to mark the core of the granuloma (**Figure 2**). In agreement with the data generated using OT-I cells transferred into mice infected with WT *L. donovani* or PINK parasites, OT-I cells had slower average velocity than F5 T cells imaged simultaneously in granulomas of PINK-infected mice (**Figure 7H, I** **and Video S6**). Thus, the presence or absence of cognate antigen determines the dynamics of CD8^+^ T cell motility in hepatic granulomas.

### CD8^+^ T cells undergo antigen-specific interactions within *L. donovani*-induced hepatic granulomas, reflecting *in vivo* presentation of cognate antigen by Kupffer cells

**Figure 8:**
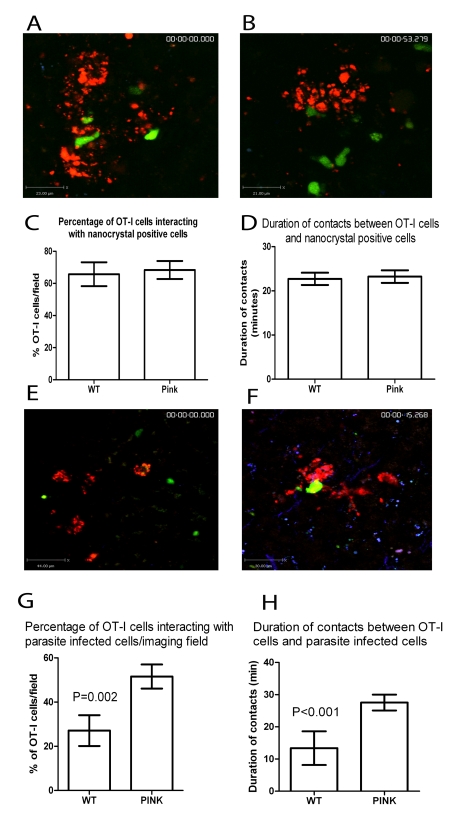
CD8^+^ T cell interactions with parasite infected cells. **A**) Snapshot of the extended focus view of a time-lapse imaging sequence showing transferred memory-like OT-I T cell (green) interactions with nanobead labelled cells (red) in the livers of d14-21 WT *L. donovani* and **B**) PINK infected mice. Quantification of **C**) the percentage and **D**) the duration of contacts between nanocrystal labelled cells and OT-I T cells in the livers of WT *L. donovani* and PINK infected mice (n = 57 OT-I cells for WT and 60 OT-I cells for PINK). **E**) Snapshot of the extended focus view of a time-lapse imaging sequence showing transferred memory-like OT-I T cell (green) interactions with amastigote infected cells (red) in the liver of d14-21 WT *L. donovani* and **F**) PINK infected mice. Quantification of **G**) the percentage and **H**) the duration of contacts between amastigote-infected cells and OT-I T cells in the livers of WT *L. donovani* and PINK infected mice, (n = 32 OT-I T cells for WT and 58 OT-I T cells for PINK). Data represents mean ± SEM.

To determine whether this antigen-dependent reduction in CD8^+^ T cell motility was due to more extensive or more prolonged interactions with granuloma-resident cells presenting MHCI-peptide complexes, we first asked whether transferred OT-I cells interacted with the granuloma-associated KCs, by labelling the latter at the onset of infection with NBs, as described above. Transferred OT-I cells were observed to make frequent contacts with NB-labelled KCs (defined by large aggregates of NBs; **Figure 8A and B** **and Video S7**). However, the presence of cognate antigen did not influence either the percentage of OT-I T cells interacting with NB^+^ cells (**Figure 8C**) or in the duration of these contacts (**Figure 8D**). On the other hand, as shown above, not all granuloma-associated KCs contained amastigotes (**Figure 1**) and similarly not all cells with this phenotype expressed detectable K^b^-SIINFEKL complexes (**Figure 4**). Many NB^+^ KC would be expected, therefore, to be devoid of antigen/parasites, and represent KCs recruited during the process of granuloma development (**Figure 2**), with the net effect of diluting out any the effect of any antigen-specific interactions between KCs and OT-I cells. Therefore, to more directly assess the potential of infected KCs to present OVA peptide, we infected mice with tdTom-PINK or tdTom-WT *L. donovani* and evaluated the interaction of these cells with CFSE-labelled transferred OT-I T cells (**Figure 8E and F** **and Video S8**). As with NB-labelled cells, OT-I T cells made multiple contacts with amastigote-infected KCs within granulomas containing both PINK and WT *L. donovani*. However, both the frequency of intra-granuloma OT-I T cells that engaged in this behaviour (**Figure 8G**) and the subsequent duration of these contacts (**Figure 8H**) was clearly influenced by the presence of cognate antigen. These studies provide the first direct evidence of intra-granuloma antigen recognition by CD8^+^ T cells and for *in situ* presentation of MHCI-restricted peptides by KCs.

## Discussion

Granulomas are well-recognised as a central feature of the pathogenesis of human [46],[47], canine [48] and experimental [49] VL, and most if not all perturbations of immune function made under experimental conditions can be related to alterations in granuloma form and function [14],[50]. Nevertheless, the processes by which these structures form around initially infected KCs and how the microenvironment they create serves to guide and focus host effector function remain poorly understood. Here, we provide the first direct *in situ* evidence that KCs serve as targets for antigen recognition by granuloma-infiltrating CD8^+^ T cells. In addition, our study, together with that of Egen and colleagues using experimental BCG infection [19], help dispel the notion of the granuloma as being a static tissue structure and reveal the intricate dynamics of lymphocytes within this unique microenvironment.

Historically, granulomatous inflammation during *L. donovani* infection has been classified on the basis of the histological response that occurs around each infected KC, providing both a quantitative means to score granuloma ‘maturation’ and a surrogate measure of the quality of the host protective response [14],[50],[51]. Our studies using fluorochrome-reporter transgenic parasites and mice, whole mount confocal and 2-photon microscopy, performed here as a prelude to the analysis of antigen presentation within granulomas, also provide new insight into some of the basic features of granuloma formation. For example, our data shows that a significant proportion of the sinusoidal KC network becomes incorporated within developing granulomas, yet at the same time even those KCs not directly engaged in the process undergo profound morphological changes indicative of activation. Such changes in morphology have been used previously *in vitro* [52] and *ex vivo* [53] as correlates of macrophage activation, but cell volume has not previously been measured *in situ*. The correlation of cell volume with increased expression of cell surface MHCII suggests that it is a true indication of macrophage activation, opening new avenues for the use of whole mount microscopy in the study of KC activation in the study of diseases such as liver injury [54] or liver regeneration following resection or transplantation [55]. Our results also confirm earlier observations [56] that some infected KCs fail, at least for many days or even weeks, to form a focus for inflammation. This marked asynchrony in granuloma development has been the subject of debate [50] and has recently been subjected to systems biology-based approaches [57],[58], but the key determinants of this response remain to be identified. In a recent study in the model organism zebrafish, macrophages infected with BCG were able to migrate out of granulomas [56]. Although migration of *L. donovani*-infected KCs might also give rise to a population of infected cells apparently uninvolved in the granuloma formation, we do not believe that this scenario is likely in the intact mammalian host, as in neither our studies nor in those of Egen et. al [19] has KC exit from granulomas been observed.

The main focus of this study, however, was on identifying the nature of the cells which engaged with effector CD8^+^ T cells within the granuloma microenvironment, and in this regard, we provide the first *in vivo* evidence of a cognate interaction between KCs and antigen-specific CD8^+^ T cells. Whereas KCs were abundant in granulomas, CD11c^hi^ F4/80^−^ DCs were notable by their relative paucity, a finding also reflected in the low frequency of CD11c^hi^F4/80^−/int^ DC observed in mononuclear cell preparations made from infected mice. Although CD11c^+^ cells were detectable, co-labelling with F4/80, the presence of high numbers of intracellular amastigotes and labelling with NBs confirmed that most of these cells were KCs on which CD11c expression had been aberrantly induced (as is also the case for DEC-205 [33]). Likewise, we observed few CD11b^+^CD11c^+^ cells in granulomas, and CD11b^+^ cells rarely contained intracellular amastigotes. These later data are in stark contrast to the situation observed in the lesions of mice infected with *L. major*, where the bulk of the amastigote load has been reported to reside within CD11b^+^CD11c^+^ ‘inflammatory monocytes’ or ‘TipDC’ [59],[60]. Our data are, however, consistent with earlier reports that indicated both a preference by *L. donovani* for infection of ‘resident’ compared to inflammatory macrophages and the greater capacity of *L. major* to stimulate CD11b^+^ cell recruitment even to hepatic sites of infection [61].

To determine the capacity of these infected KCs to interact in a cognate manner with effector CD8^+^ T cells, we first tried using immunohistochemical approaches and flow cytomtery to identify which cells could process SIINFEKL from OVA-transgenic PINK parasites and then form complexes recognised by mAb 25-D1.16 [38]. We were unable to detect expression by any immunohistochemical approach we tested (including teramide labelling). By flow cytometry, however, we could detect specific staining on CD11c^int^F4/80^int^ cells that we believe represent intra-granuloma KCs. Such staining was notably absent on CD11c^hi^F4/80^−/int^ DCs. These *ex vivo* analyses should however be viewed with some caution. First, granulomas cannot be specifically isolated for analysis, and as a consequence cells analysed by flow cytometry may originate from any anatomical compartment within the infected liver. Second, we cannot exclude the possibility that MHCI-peptide complexes and/or whole parasites are either shed or transferred to other cells during the isolation procedure. Such transfer of MHCI-peptide complexes has been noted under *in vitro* culture conditions [62],[63] and indeed transfer of parasites between populations of cells during tissue disruption and subsequent cell isolation has been noted by us (Figure S1) and by others [59]. It is, however unlikely that processing of antigen into MHCI is able to occur within the 30 min collagenase digestion step, or the 10 min density gradient centrifugation steps both of which were performed at room temperature, as detection of MHC-I-peptide complexes takes >1 hr following virus infection [64] and is likely to follow similar kinetics following *Leishmania* infection. All other processing steps were performed on ice. Whilst analysis of the expression of MHCI-peptide complex expression might, therefore, also suffer from the same technical difficulties, we believe this is unlikely. Third, only low numbers of MHCI-peptide complex are required for productive engagement with CD8^+^ T cells [65], well below that detectable by mAb staining. These caveats notwithstanding, our data suggested that KCs and not DCs expressed such complexes in most abundance. It should also be noted that sessile KCs are not readily isolated by the methods we used [5] and as such have been largely excluded from this *ex vivo* analysis. We cannot therefore comment on whether such KCs do or do not express complexes recognised by 25-D1.16.

To more definitively identify the sites of antigen presentation, we therefore turned to intra-vital imaging of adoptively transferred CD8^+^ effector cells. CD62L^lo^ effector CD8^+^ T cells generated in vitro using antigen expansion and IL-2, were chosen for analysis, as these cells have previously been shown to bring about a rapid and antigen-specific reduction in hepatic parasite burden [25]. Furthermore, analysis of the fate of such cells may provide clues as to how similar effector cells induced by vaccination may behave. We used similar methods to those of others working in lymphoid tissue [66],[67], in tumor microenvironments [68],[69], in BCG granulomas [19] and in the brains of *Toxoplasma gondii* infected mice [70],[71] to define the dynamic behaviour of CD8^+^ T cells in hepatic granulomas caused by *L. donovani* and our results not surprisingly showed marked similarities in T cell behaviour. The dynamic nature of the T cell compartment within the *L. donovani* granuloma was also clearly evident in all the imaging that we performed. Though superficially similar to that reported for BCG infection, contrasts between *L. donovani* and BCG granulomas can be noted. For example, whilst T cells were reported to stay within the granuloma structure following BCG injection [19], we found that endogenous T cells, as well as adoptively transferred antigen-specific and non-antigen specific CD8^+^ T cells, could readily migrate out of granulomas, indicating a net flux through this ‘compartment’. Additionally, while a marked difference in the velocity of cells tracked within and outside of BCG induced granulomas showed that the granuloma per se was capable of inducing a change in cell movement [19], CD8^+^ T cells migrated without apparent constraint into, within and out of *L. donovani* granulomas, and non-antigen specific cells showed the same speed of cell movement whether located inside or outside of granulomas. Similarly, analysis of the instantaneous velocity of cells within and outside of granulomas showed no obvious differences, confirming the presence of antigen as the only factor that induces a change in cellular behaviour. The differences in behaviour of T cells in these two types of granuloma may be attributable to differences in composition of the mononuclear cell mantle or reflect differences in other environmental factors e.g. the level of fibrosis [43],[44]. The traffic of antigen-non-specific T cells through granulomas also provides a timely reminder that the histological identification of T cells within granulomas, in the absence of dynamic measurement, is neither an indicator of antigen-specificity nor a good marker for the effector capacity of these structures.

In most cases, we validated our approach by cross-over experiments in which on the one hand we used adoptive transfer of OT-I T cells into mice infected with WT *L. donovani* or PINK parasites, and on the other hand, we used co-transfer of OT-I and F5 T cells into PINK-infected mice. While labelling with NBs was sufficient to allow the identification of the core of the granuloma, it does not delimit the extent of the granuloma. Hence, CD8^+^ T cells often appear distant from the core of the granuloma, they were still maintained within its boundaries. Cells frequently migrated near to and, in fact, through the NB-labelled core, but the interactions with NB-labelled cells were not sufficient to demonstrate antigen-specific interactions. This result is not surprising, given that not all cells present in the granuloma core contained parasites (Figure 1) and the effect of any antigen-specific contacts with infected NB^+^ cells would likely be diluted out by interactions with non-infected NB^+^ cells. Meandering of antigen-specific CD8^+^ T cells was extensive, as might be predicted from the dense packing of lymphocytes within granulomas and whereas migration upon collagen fibres was noted, this was not seen in all instances. Numerous contacts were also made between CD8^+^ T cells and amastigote-infected KCs. Importantly, as measured by all these parameters, the intra-granuloma behaviour of effector CD8^+^ T cells was markedly influenced by the presence of cognate antigen.

In spite of clear data supporting intra-granuloma antigen recognition by CD8^+^ T cells, we have not to date observed evidence of direct effector activity of these transferred CD8^+^ T cells, such as dispersal of amastigotes or their loss of apparent viability. The failure to do so may be due to the length of time taken for CTL to lyse targets *in vivo*, with target cell lysis *in vivo* reported to take as little as 17 min in the case of target cells pulsed with high doses of peptide [72] or as long as 6 hours for tumour targets [73]. Additionally, there are technical limitations to these methods as the maximum imaging window we can achieve is 10 h, and this may be insufficient to observe degradation of amastigotes (and/or tdTom protein) subsequent to cytokine-mediated macrophage activation. Similarly, as KC integrity was not directly imaged in these experiments, it is possible that host cell lysis occurs but amastigotes were rapidly re-engulfed by neighbouring KCs. Furthermore, we cannot rule out that after recognition, CD8^+^ T cells exert their leishmanicidal effect indirectly and over a longer time frame than examined here. Additional developments in imaging technology and new tools to study macrophage responsiveness to activation signals in real time will be required to conclusively address this issue. Although our data are the first to directly demonstrate KC interactions with effector CD8^+^ T cells, KC-mediated priming of CD8^+^ T cells was recently demonstrated using cell lines *in vitro* [74] and also with freshly isolated KCs *ex vivo* [75], suggesting that further studies into the role of KC in presenting *Leishmania*-derived antigens to naïve CD8^+^ T cells at the initiation of infection are also now warranted.

In conclusion, we have shown that KCs laden with amastigotes serve as the principal target for antigen recognition by effector CD8^+^ T cells within the granuloma microenvironment. Our data suggest that if CD8^+^ T cell recognition is to form the basis for prophylactic or therapeutic vaccination, then it will be essential to understand the rules which govern MHC class I epitope selection within infected KCs, as well as within those APC (e.g. DCs) that are responsible for induction of CD8^+^ T cell responses. Furthermore, chemotherapeutic or immunotherapeutic interventions that enhance antigen presentation by KCs may prove highly beneficial.

## Methods

### Mice and infection

C57BL6 mice were obtained from Charles River (UK). hCD2.GFP [42] and VaDS Red B6 and Rag-1^−/−^ F5 mice, originally a kind gift from Dimitris Kioussis (NIMR, Mill Hill, UK), and Rag-1^−/−^ OT-I mice were bred and housed under specific pathogen-free conditions and used at 6–12 weeks of age. The Ethiopian strain of Leishmania donovani (LV9) and OVA expressing LV9 (PINK) [25] were maintained by serial passage in Rag-1^−/−^ mice. Amastigotes were isolated from infected spleens, as previously described [24], and mice were infected with 2×10^7^ *L. donovani* amastigotes intravenously (i.v.) via the tail vein in 200 µl of RPMI 1640 (GIBCO, Paisley, UK). For pre-labelling of liver-resident macrophages, PD nanobeads (545 marked) (Sigma) were pre-injected into mice i.v. 5–24 hours prior to injection of *L. donovani* amastigotes. All experiments were approved by the University of York Animal Procedures and Ethics Committee and performed under UK Home Office license (‘Immunity and Immunopathology of Leishmaniasis’ Ref # PPL 60/3708).

### Generation of tdTom *L.donovani*

Tandem Tomato fluorescent protein (tdTom) gene [30] was cloned into the plasmid pSSU-Neo-Infantum to give pSSU-Neo-Infantum-tdTom [76] [Oyola et. al. manuscript in prep]. WT *L. donovani* and *L. donovani* HASPB::OVA (PINK) promastigotes [25],[36] were transfected with this construct (which targets genes into the ribosomal locus of *L. donovani*) and clones selected by serial dilution in the presence of neomycin. Clones were checked for correct integration of the tdTom gene by PCR and Southern Blotting of *Bam*HI and *Sca*I digested genomic DNA with a 586 bp probe against the neomycin phosphotransferase gene.

### Confocal microscopy

Confocal microscopy was performed on 8–10 µm frozen sections. For tissue containing tdTom expressing parasites, tissue was fixed in 4% paraformaldehyde (PFA) for two hours before overnight incubation in 30% sucrose and embedding in Optimal Cutting Temperature (OCT) medium (Sakura). For all other labelling, tissue was snap-frozen in OCT and sections fixed in ice cold acetone for 8 min. F4/80, CD11c and CD11b antibodies were conjugated to Alexa488 or Alexa647 (eBioscience, UK) and Rabbit anti-desmin (Abcam) was detected with goat-anti Rabbit-647 (Invitrogen).

For whole mount confocal microscopy, thick tissue sections were cut with a scalpel blade and labelled as previously described [77]. Briefly, sections were fixed in 4% PFA for 15 min at room temperature (RT), washed in PBS-Triton (0.15%) and blocked for 2 hours at RT. All subsequent antibody labelling steps were performed for 8 hours or overnight at 4°C followed by final fixing in 4% PFA for 15 min at RT followed by dehydration in methanol. Samples were optically cleared in BABB (sigma) and imaged using a Zeiss LSM510 axioplan microscope (Carl Zeiss Microimaging). Data were rendered and analysed using Volocity software (Improvision). Cell volumes were calculated by generating a measurement item based on RGB and exclusion of objects <300 µm^3^ and >15 0000 µm^3^. All objects were manually checked for accuracy before data were plotted and analysed in Prism v5.1 (Graphpad).

### Flow cytometry and cell sorting

Hepatic mononuclear cells were prepared from the livers of wild type and PINK *L. donovani* infected livers, or livers from C57BL/6 mice injected with 100 µg SIINFEKL peptide I.V, following collagenase digestion as previously described [78]. Briefly, livers were perfused with PBS containing 2%FCS and digested in 350 µg/mL collagenase D (Worthington, UK) for 30 min at RT. Digested livers were passed through a 100 µm cell strainer, washed twice in 2%FCS.PBS and hepatocytes removed by centrifugation on a 33% percoll density gradient for 12 min at 693 g. The remaining cell pellet was kept for further analysis. Isolated cells were labelled with 25-D1.16-biotin [38] and streptavidin-Alexa488 as well as CD11c-PeCy7, F4/80-Alexa647 and CD11bPE or pacific blue (eBioscience, UK). Cells were sorted based on expression of tdTom, with approximately 3000 sorted cells spun onto glass slides, fixed in methanol and stained with Giemsa for morphological analysis.

### OT-I transfers

CD8^+^ T cells resembling effector memory cells were derived *in vitro* as described previously [45]. Briefly, splenocytes from naive OT-1 transgenic mice were incubated with 10 µg/ml OVA_257–264_ (Cambridge Bioscience) for 1 h at 37°C, washed, and cultured for a further 48 h. Cells were then washed and incubated for a further 5–9 days with 20 ng/ml recombinant hIL-2. CD62-L low cells were enriched to >95% purity by negative selection using anti-CD62-L microbeads (Miltenyi). Enriched cells were labeled with 5 µM CFSE or CMTMR (invitrogen) or 6 µM Hoescht 33342 (Sigma) before transfer of 2×10^7^ cells to recipient mice by intravenous injection.

### 2-photon imaging of fresh explanted tissue

Freshly removed liver tissue was placed in 35 mm coverslip bottom Petri dishes (MatTek corporation), kept moist with PBS and imaged on an inverted LSM 510 multiphoton microscope (Carl Zeiss Microimaging). Images were acquired with a 40×1.1 water immersion objective and fluorescence excitation provided by a Chameleon XR Ti:sapphire laser (Coherent) tuned to 872 nm. Data were rendered and analysed using Volocity software (Improvision). Granuloma volumes were calculated by drawing regions of interest in Volocity to get a 3D volume measurement in µm^3^. Exported videos were arranged in After Effects software (Adobe).

### Intravital imaging

Mice were anaesthetised with a combination of ketamine (100 mg/kg), xylazine (10 mg/kg) and acepromazine (1.7 mg/kg) given intraperitoneally. After 60 min, anaesthesia was maintained by subcutaneous injections of half doses approximately every 45 min. The abdomen of the animal was shaved, and a ∼1.5 cm midline incision made to expose the xiphoid process which was retracted to allow dissection of the falciform ligament. The left lobe of the liver was then gently exteriorised and the animal inverted onto a glass coverslip mounted within a custom made imaging platform. The liver was covered with sterile saline-soaked gauze to prevent dehydration and the mouse stabilised with micropore tape (3 M). Images were acquired on an inverted LSM 510 multiphoton microscope (Carl Zeiss Microimaging) (as above) which was maintained at 36°C by a blacked-out environmental chamber (Solent Scientific, UK). For 4D analysis, 20–35 µM Z stacks were acquired with a Z distance of 2–3 µM approximately every 15–30 sec. Data were rendered and analysed using Volocity software (Improvision) and cell tracking performed manually, or automatically with manual checking. Entrance and exit rates were calculated by monitoring the number of OT-I cells entering or exiting granulomas, as defined by the endogenous T cell border or the presence of nanocrystals within each imaging window and dividing this number by the time of each imaging window in minutes, to get a rate of OT-I entrance and exit per minute. All granulomas imaged were included in this analysis, irrespective of whether they had associated OT-I cells.

## Supporting Information

Figure S1 Release of free amastigotes prevents the use of tdTom as a tag to track infected cells *ex vivo*. A)-C) C57BL/6 mice were infected with tdTom-*L donovani* amastigotes and the hepatic mononuclear cells prepared into a single cell suspension. A) Cells were sorted based on forward and side scatter and B) expression of tdTom. C) Sorted cells were spun onto glass slides, fixed in methanol and Giemsa stained, showing that sorted cells had morphology consistent with macrophages, monocytes, polymorphonuclear cells and lymphocytes but numerous parasites were stuck to the outside of cells. D)-F) B6.CD45.1 mice were infected with tdTom-*L. donovani* amastigotes and 28 days later the hepatic mononuclear cells prepared, after adding the liver from a day 14 WT-L. donovani infected C57BL/6 (CD45.2) mouse into the same preparation. D) Hepatic mononuclear cells from a WT-*L donovani* infected mouse as a control for the tdTom gating. E) tdTom positive cells were gated from the mix of the tdTom-*L. donovani* CD45.1 infected cells and the WT-*L. donovani* infected CD45.2 cells. F) CD45.2 and CD11b expression from tdTom positive cells from E) showing that transfer of parasites occurs from CD45.1 to CD45.2 cells during *ex vivo* preparation.
(5.25 MB TIF)

Figure S2 Recruitment of mononuclear cells 4–12 hrs post-infection with *L. donovani* amastigotes. C57BL/6 mice were injected with PD nanobeads 12 hr prior to infection with 3×10^7^ *L. donovani* amastigotes. Six hours later, hepatic mononuclear cells were prepared from A) mice that received nanocrystals only or B) mice that received both nanocrystals and parasites. Cells were labelled with CD11b to determine the C) percentage and D) absolute number of CD11b negative, intermediate and high cells from mice that received nanobeads only (white bars) or nanobeads and *L. donovani* (grey bars). Data in A) and B) is representative of 5 mice per group. C) and D) show the mean +/− SEM for 5 mice per group.
(2.15 MB TIF)

Figure S3 The effect of the *L. donovani* granuloma microenvironment on CD8^+^ T cell movement. Analysis of the instantaneous velocities of cells contained within Video S6. Individual cells from WT *L. donovani* infected mice are shown: A) inside the granuloma, represented by the purple track; B) inside the granuloma, represented by the green track; C) outside of the granuloma (top right), represented by the yellow track; and D) outside of the granuloma (top right), represented by the orange track.
(0.79 MB TIF)

Video S1 Kupffer cell distribution in naïve and *L. donovani* infected liver. 3D projections of F4/80^+^ Kupffer cells (green) as detected by whole mount immunohistochemistry showing the distribution in naïve (left) and *L. donovani* infected (right) livers.
(3.71 MB AVI)

Video S2 *L. donovani* hepatic granulomas in 3 dimensions. Three examples of the T cell structure seen in *L. donovani* hepatic granulomas in hCD2.GFP mice (T cells green) 14d after infection. The collagenous liver capsule is seen as a result of second harmonic generation (blue).
(6.18 MB AVI)

Video S3 Distribution of amastigotes in the liver following *L. donovani* infection. Three examples of the distribution of amastigotes (red) 14 days after infection of hCD2.GFP reporter mice (T cells green) with TdTom *L. donovani*. The collagenous liver capsule is seen as a result of second harmonic generation (blue).
(3.13 MB AVI)

Video S4 Antigen specific accumulation of CD8^+^ T cells within *L. donovani* granulomas. 3D view of Z-stacks collected from the livers of d21 OVA expressing PINK *L. donovani* infected hCD2.GFP reporter mice (T cells green) that received 10^7^ memory-like CMTMR labelled OT-I T cells (red) 4 hours (left) or 12 hours previously (right). The collagenous liver capsule is seen as a result of second harmonic generation (blue). Arrows showing the classification of OT-I T cells considered within and outside of granulomas are shown at 11 sec.
(5.89 MB AVI)

Video S5 CD8^+^ T cell dynamics within *L. donovani* hepatic granulomas. Extended focus views of time-lapse image sequences showing the rapid migration of endogenous T cells (red) and OT-I T cells (green) in hepatic granulomas from d14–21 infected hCD2.DsRED mice. Cell tracks of the OT-I T cells are shown as overlays, the collagen structure of the liver is seen as a result of second harmonic generation (blue).
(3.55 MB AVI)

Video S6 Dynamics of F5 and OT-I T cell movement within *L. donovani* hepatic granulomas. Extended focus views of time-lapse imaging sequences showing the migration of Hoechst labelled OT-I T cells (blue, blue tracks) and CFSE labelled F5 T cells (green, green tracks) following transfer into a d14 OVA expressing PINK *L. donovani* infected mouse that was pre-injected with nanocrystals to label tissue resident macrophages (red).
(3.69 MB AVI)

Video S7 Interactions between CD8^+^ T cells and tissue resident macrophages at the core of *L. donovani* hepatic granulomas. Extended focus views of time-lapse imaging sequences showing interactions between CFSE labelled OT-I T cells (green) and tissue resident macrophages (red) following transfer of 10^7^ OT-I T cells into d14-21 wild type (left) or OVA expressing PINK (right) *L. donovani* infected mice that were pre-injected with nanocrystals.
(2.21 MB AVI)

Video S8 Interactions between CD8^+^ T cells and parasite infected cells. Extended focus views of time-lapse imaging sequences showing interactions between CFSE labelled OT-I T cells (green) and *L. donovani* infected cells (red) following transfer of 10^7^ memory-like OT-I T cells into d14-21 wild type tdTom *L. donovani* (left) or OVA expressing PINK TdTom *L. donovani* (right) infected mice.
(2.92 MB AVI)
