# Supplementary figures and images for: Dynamic Imaging of Experimental Leishmania donovani-Induced Hepatic Granulomas Detects Kupffer Cell-Restricted Antigen Presentation to Antigen-Specific CD8+ T Cells
Source: PLoS Pathog. 2010 Mar 12;6(3):e1000805. doi: 10.1371/journal.ppat.1000805 (PMC2837408; doi:10.1371/journal.ppat.1000805)

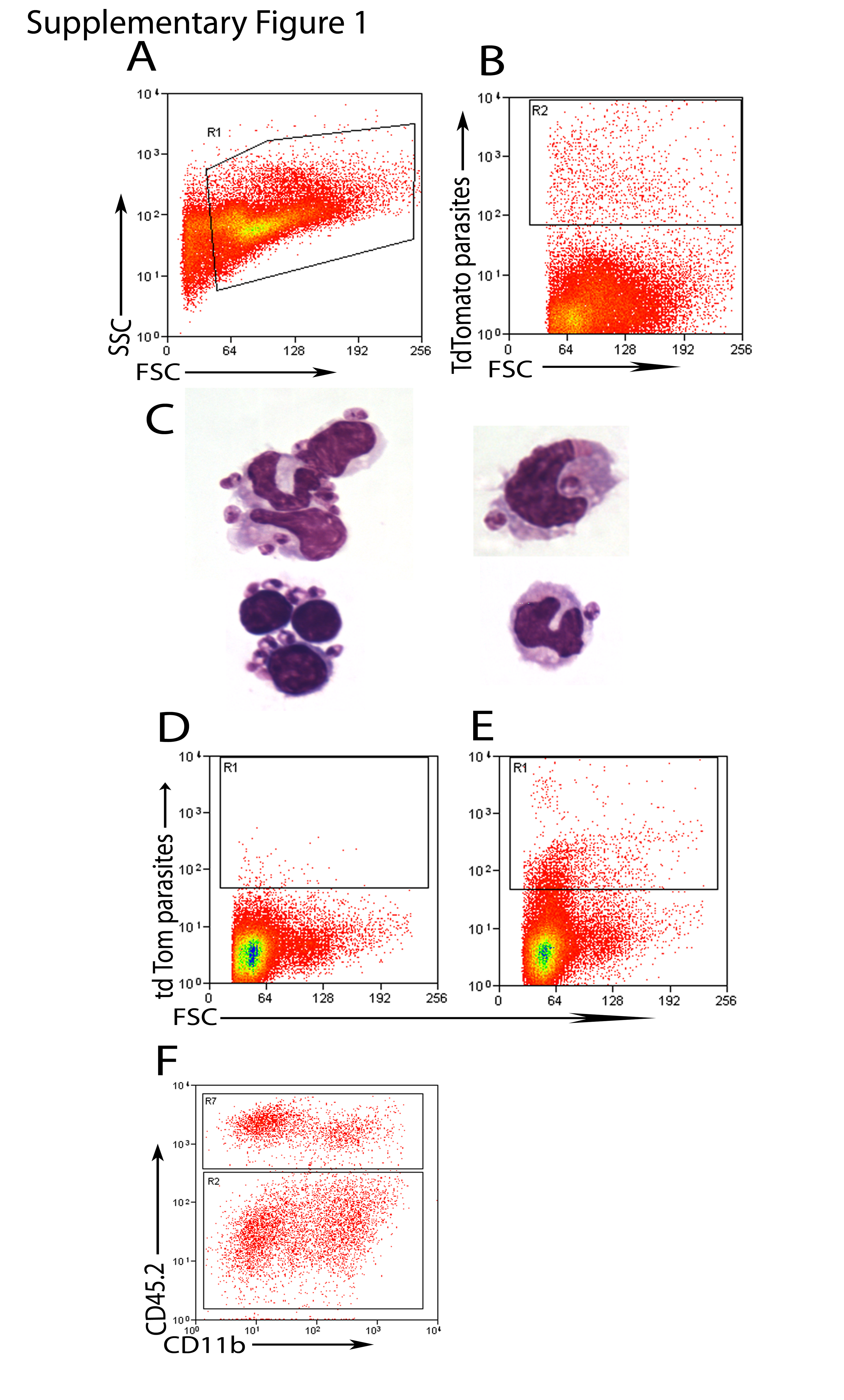

Supplement: Figure S1 — Release of free amastigotes prevents the use of tdTom as a tag to track infected cells ex vivo. A)-C) C57BL/6 mice were infected with tdTom-L donovani amastigotes and the hepatic mononuclear cells prepared into a single cell suspension. A) Cells were sorted based on forward and side scatter and B) expression of tdTom. C) Sorted cells were spun onto glass slides, fixed in methanol and Giemsa stained, showing that sorted cells had morphology consistent with macrophages, monocytes, polymorphonuclear cells and lymphocytes but numerous parasites were stuck to the outside of cells. D)-F) B6.CD45.1 mice were infected with tdTom-L. donovani amastigotes and 28 days later the hepatic mononuclear cells prepared, after adding the liver from a day 14 WT-L. donovani infected C57BL/6 (CD45.2) mouse into the same preparation. D) Hepatic mononuclear cells from a WT-L donovani infected mouse as a control for the tdTom gating. E) tdTom positive cells were gated from the mix of the tdTom-L. donovani CD45.1 infected cells and the WT-L. donovani infected CD45.2 cells. F) CD45.2 and CD11b expression from tdTom positive cells from E) showing that transfer of parasites occurs from CD45.1 to CD45.2 cells during ex vivo preparation. (5.25 MB TIF) [file ppat.1000805.s001.tif]

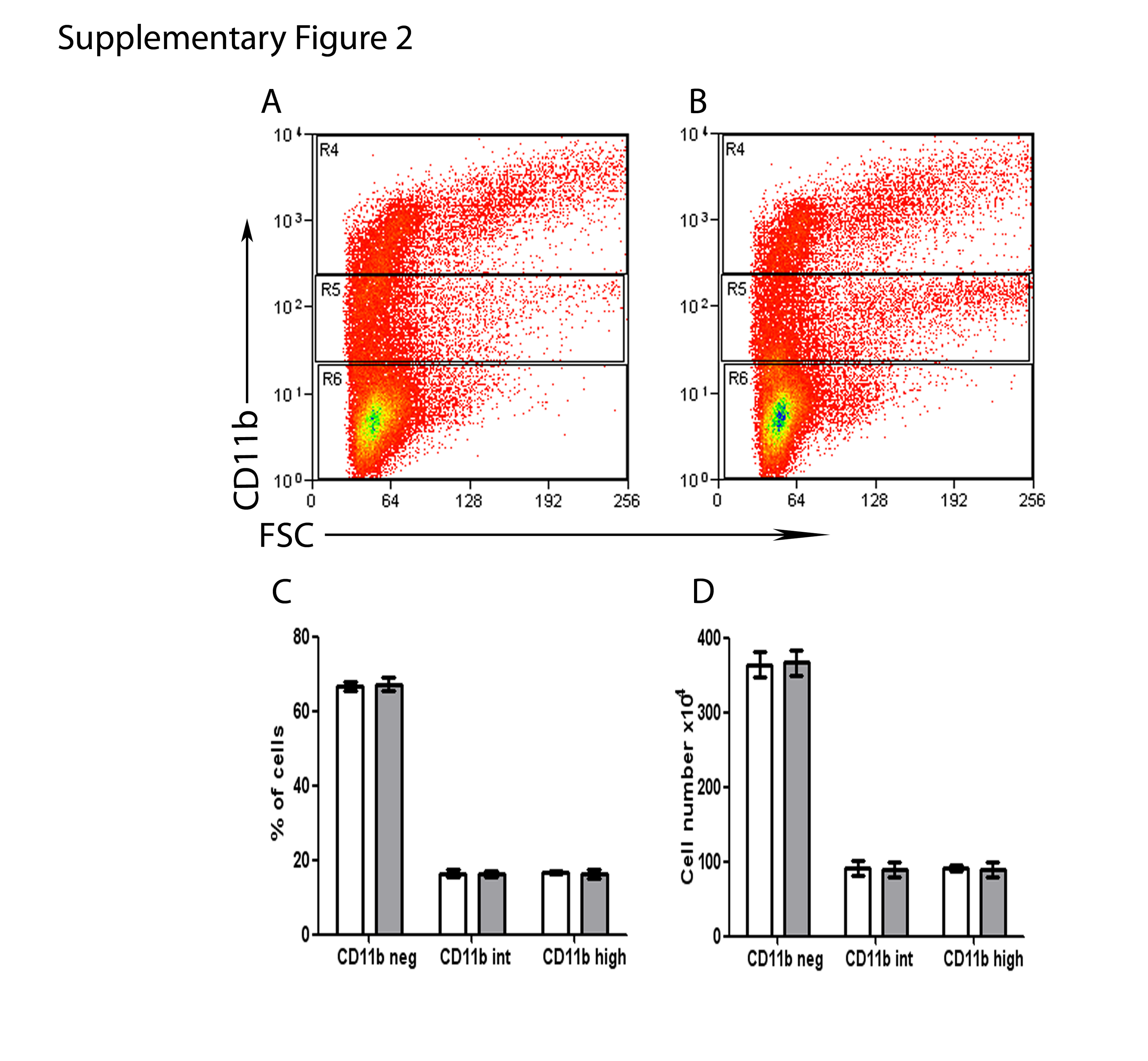

Supplement: Figure S2 — Recruitment of mononuclear cells 4–12 hrs post-infection with L. donovani amastigotes. C57BL/6 mice were injected with PD nanobeads 12 hr prior to infection with 3×10^7^ L. donovani amastigotes. Six hours later, hepatic mononuclear cells were prepared from A) mice that received nanocrystals only or B) mice that received both nanocrystals and parasites. Cells were labelled with CD11b to determine the C) percentage and D) absolute number of CD11b negative, intermediate and high cells from mice that received nanobeads only (white bars) or nanobeads and L. donovani (grey bars). Data in A) and B) is representative of 5 mice per group. C) and D) show the mean +/− SEM for 5 mice per group. (2.15 MB TIF) [file ppat.1000805.s002.tif]

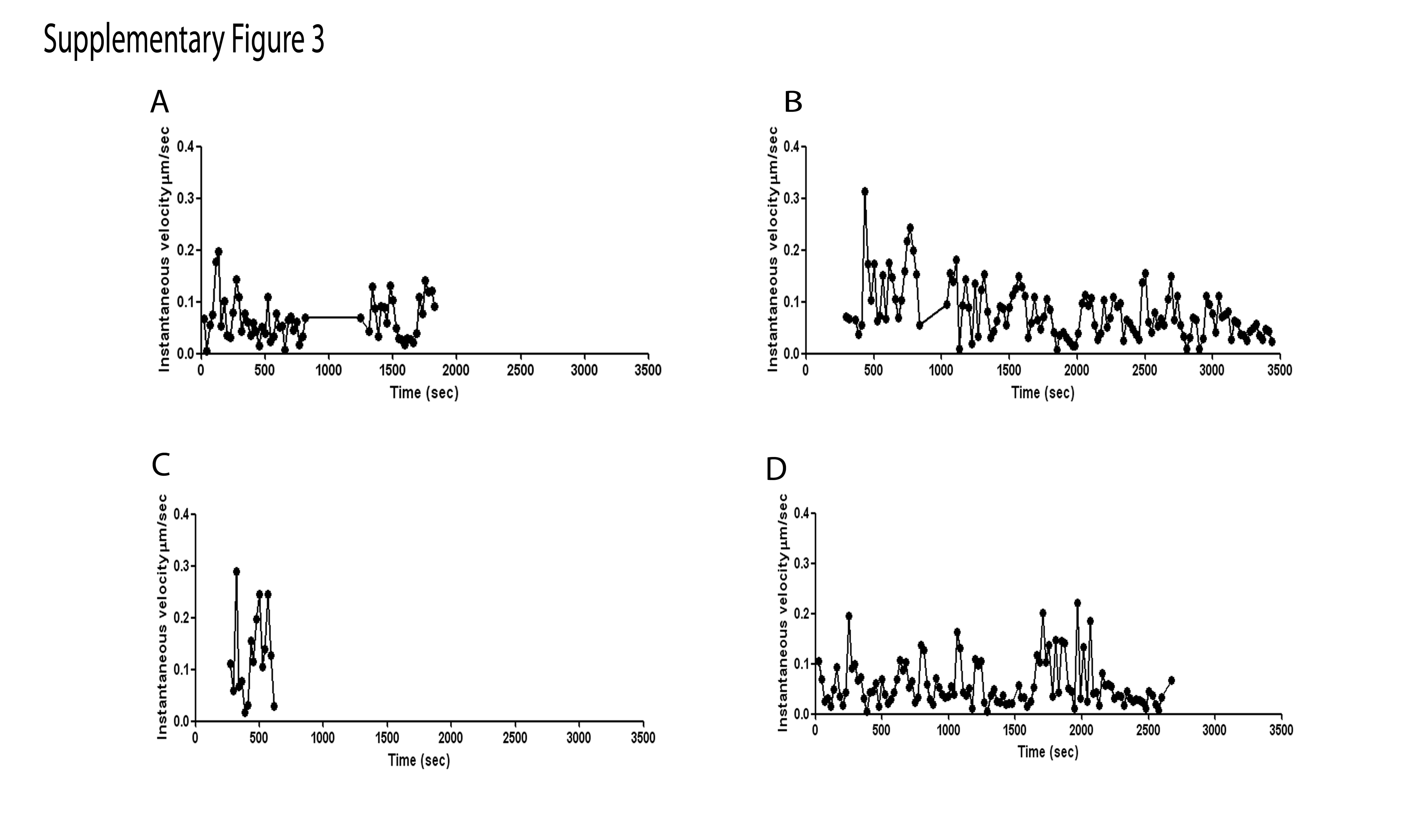

Supplement: Figure S3 — The effect of the L. donovani granuloma microenvironment on CD8+ T cell movement. Analysis of the instantaneous velocities of cells contained within Video S6. Individual cells from WT L. donovani infected mice are shown: A) inside the granuloma, represented by the purple track; B) inside the granuloma, represented by the green track; C) outside of the granuloma (top right), represented by the yellow track; and D) outside of the granuloma (top right), represented by the orange track. (0.79 MB TIF) [file ppat.1000805.s003.tif]
